# Experimental Investigation on Thermal Performance Optimization of Na_2_HPO_4_·12H_2_O-Based Gel Phase Change Materials for Solar Greenhouse

**DOI:** 10.3390/gels11060434

**Published:** 2025-06-05

**Authors:** Wenhe Liu, Gui Liu, Wenlu Shi, Xinyang Tang, Xuhui Wu, Jiayang Wu, Zhanyang Xu, Feng Zhang, Mengmeng Yang

**Affiliations:** 1 College of Ocean and Civil Engineering, Dalian Ocean University, Dalian 116023, China; liuwenhe@dlou.edu.cn; 2 College of Engineering, Shenyang Agricultural University, Shenyang 110866, China; lg17603200141@stu.syau.edu.cn (G.L.); 2024200013@stu.syau.edu.cn (W.S.); 2023240210@stu.syau.edu.cn (X.T.); 2024240175@stu.syau.edu.cn (X.W.); 2022166086@stu.syau.edu.cn (J.W.); xuzhanyang@syau.edu.cn (Z.X.); fzhang2019@syau.edu.cn (F.Z.)

**Keywords:** phase changing material, Na_2_HPO_4_·12H_2_O-based, Taguchi method, solar greenhouse

## Abstract

The content of modified materials in multicomponent gel phase change materials directly affects their performance characteristics. To investigate the influence of different contents of modified materials on the performance features of Na_2_HPO_4_·12H_2_O-based multicomponent Gel Phase Change Materials, four single factors (Na_2_SiO_3_·9H_2_O, C_35_H_49_O_29_, KCl, and nano-α-Fe_2_O_3_) and their interactions were selected as influencing factors. Using the Taguchi method with an L_27_(3^13^) orthogonal array, multi-step melt–blending experiments were conducted to prepare a novel multi-component phase change material. The characteristics of the new multi-component phase change material, including supercooling degree (ΔT), phase change temperature (T_m_), latent heat of phase change (ΔH_m_), and cooling time (CT), were obtained. In addition, characterization techniques such as DSC, SEM, FT-IR, and XRD were employed to analyze its thermal properties, microscopic morphology, chemical stability, and crystal structure. Based on the experimental results, the signal-to-noise ratio (S/N) was used to rank the influence of each factor on the quality characteristics, and the *p*-value from analysis of variance (ANOVA) was employed to evaluate the significance of each factor on the performance characteristics. Then, the effects of each significant factor on the characteristics of the multiple gel phase change materials were analyzed in detail, and the optimal mixing ratio of the new multiple gel phase change materials was selected. The results showed that Na_2_SiO_3_·9H_2_O, KCl, and α-Fe_2_O_3_ were the most critical process parameters. This research work enriches the selection of composite gel phase change materials for solar greenhouses and provides guidance for the selection of different modified material contents using Na_2_HPO_4_·12H_2_O as the starting material.

## 1. Introduction

Rapid economic development was accompanied by rapid growth in energy consumption. According to the statistics of the International Energy Agency, as of 2019, 81% of the global primary energy supply came from these fossil fuels, which is a shocking proportion. Fossil fuels, the driving force of human social development, still dominated the global energy mix [1], and the proportion of building energy consumption had been rising over the past few years, now reaching 40%, and is expected to increase by 28% in 2035 [2]. In response to climate change, countries around the world reached a consensus on environmental actions, set annual targets for carbon and silicon emissions and carbon neutrality [3], and set off a global wave of green and low-carbon transformation. Developing new energy sources and conserving energy were essential pathways. Thermal energy storage technology (TES) was a promising and realistic way to improve energy efficiency [4].

TES was capable of storing solar energy and excess electrical heat generated in buildings and industrial machines. This technology solved the problem of thermal energy utilization which was limited by space and time and bridged the gap between energy supply and energy demand [5]. TES mainly consists of sensible heat storage, chemical storage, and latent heat storage. Among them, phase change materials (PCMs) had been developed for latent heat storage [6]. When the ambient temperature rose to the melting point of the material, the solid PCMs started to melt and absorbed a large amount of heat energy. When the ambient temperature dropped to the solidification point of the material, the liquid phase change material started to solidify, released the absorbed heat energy in the process, and maintained the stability of the ambient temperature. PCMs had a stable phase change process, a high latent heat density, and good safety and controllability [7].

Scholars had extensively studied phase change energy storage and PCM for solar greenhouse applications. Specifically, this research involved the design and development of TES and PCMs [8,9,10,11], encapsulation of PCM and incorporation into building materials [12,13,14]. In the field of solar greenhouses, PCMs could mainly be embedded into the surrounding walls. Beyhan et al. [15] applied PCM to greenhouses and achieved energy savings of up to 47 kW in 28 days. Ling et al. [16] continuously monitored PCMs for 61 days and showed that sunny weather had helped to improve the efficiency of PCM compared to cloudy weather. Therefore, PCM-based TES technology will play a crucial role in the future thermal management of solar greenhouses.

Most of the previous research focused on the application of organic PCMs in the field of solar greenhouses [17,18,19,20]. However, organic PCMs had defects such as low enthalpy of phase change [21], high material cost [22], and poor thermal conductivity [23,24], which seriously hindered their application in solar greenhouse thermal management materials. Compared with organic PCMs, inorganic PCMs had higher thermal storage performance, higher thermal conductivity, were non-flammable, green and non-toxic, and had lower cost [25,26,27,28]. However, inorganic salts suffered from drawbacks such as high supercooling, easy phase separation [29], and phase transition temperatures that did not match the usage scenarios, which limited their applications [30]. The addition of nucleating agents to trigger crystal crystallization helped to reduce the degree of supercooling [31]. In hydrated salt phase change materials, thermostats were often added to weaken the interaction between salt and water molecules in hydrated salts to lower the melting point [32]. In order to suppress the phase separation problem, it was solved by adding thickeners [33,34]. Nanoparticles with nano-size effect, large specific surface effect, and strong interfacial interactions were incorporated into the phase change materials to enhance the thermal properties of the phase change materials [35]. Tang et al. [36] used Na_2_SiO_3_·9H_2_O as a nucleating agent to inhibit the supercooling of PCM, and discussed the effect of the nucleating agent on the supercooling property of PCMs. Fu et al. [37] incorporated KCl into the phase change system to regulate the phase change temperature; and the phase change temperature of the modified mixtures was significantly lower than that of the original material, and decreased with the increase of KCl mass fraction. Lan et al. [38] investigated a new gelation method for the polymerization of (C_6_H_7_NaO_6_)_n_ grafted with (C_3_H_3_NaO_2_)_n_ in Na_2_HPO_4_·12H_2_O molten salt, and confirmed that the new method could effectively eliminate the phase separation by a short-term thermal cycling. Wang et al. [39] explored the effect of the incorporation of unused masses of nano-α-Fe_2_O_3_ on the rate of thermal conductivity of the phase change materials. The results showed that nano-α-Fe_2_O_3_ could effectively increase the thermal conductivity of Na_2_HPO_4_·12H_2_O, and the addition of 0.2% nano-α-Fe_2_O_3_ increased the thermal conductivity by 90.8%.

In this paper, firstly, the experimental design was carried out by Taguchi design method, and the performance of multifunctional phase change materials with different modified material contents were obtained using Na_2_HPO_4_·12H_2_O(DHPD) as the base material, Na_2_SiO_3_·9H_2_O(SMN) as the nucleating agent, C_35_H_49_O_29_ (XG) as the additive to eliminate the phase separation phenomenon, KCl as the temperature modifier, and α-Fe_2_O_3_ as the thermal conductivity enhancer. Secondly, the effects of different modified material contents on the performance characteristics of multivariate PCMs were analyzed in detail using the S/N ratio and ANOVA methods, and the optimal mixing ratios were selected. The results showed that the poly-PCMs prepared by the selected modified materials had excellent performance. Therefore, the prepared multifunctional PCMs were promising new material.

## 2. Results and Discussion

**Figure 1:**
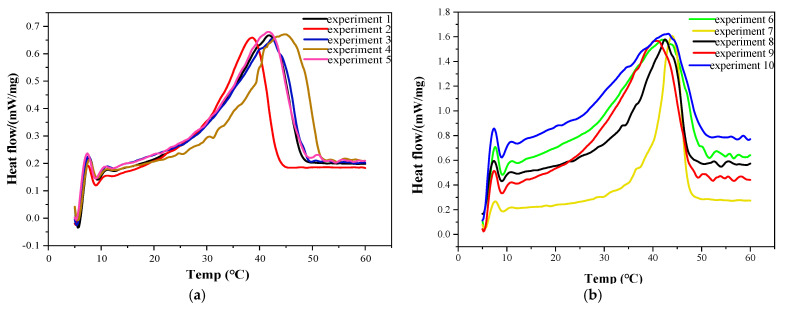

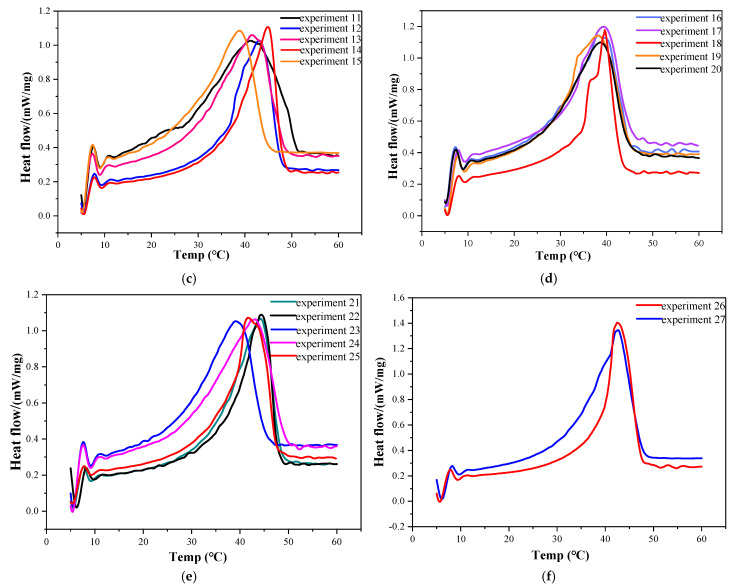
The DSC curves of 27 experimental groups: (**a**) the DSC curves of experiments 1 to 5; (**b**) the DSC curves of experiments 6 to 10; (**c**) the DSC curves of experiments 11 to 15; (**d**) the DSC curves of experiments 16 to 20; (**e**) the DSC curves of experiments 21 to 25; (**f**) the DSC curve of experiments 26 and 27.

Through a series of tests and data processing, the DSC of different components are shown in Figure 1. The experimental results of the supercooling degree (ΔT), phase transition temperature (T_m_), latent heat of phase transition (ΔH_m_), and cooling time (CT) were obtained as shown in Table 1. Then, the cooling time, and the properties of the multivariate gel phase change materials prepared with the addition of several modified materials were analyzed using signal-to-noise ratio analysis and ANOVA. These parameters, including cooling time (CT), phase transition temperature (T_m_), latent heat of phase change (ΔH_m_), and supercooling degree (ΔT), play crucial roles in evaluating the performance of the gel phase change materials.

### 2.1. Performance Characterization of PCMs with Different Content of Modified Materials

#### 2.1.1. Cooling Time Analysis

The influence of different modified material contents on the cooling time (CT) was studied through the S/N ratio, and the results are shown in Table 2. First, the primary and secondary sequential effects of each factor on each characteristic were determined based on the delta value; the larger the delta value of a factor, the greater the effect of the factor on the characteristic. The order of influence of each factor on the thermal conductivity is A > A × B > B × C > B × D > A × D > A × C > D > C × D > C > B. To further investigate the influence of the material content on each characteristic, the ANOVA method was used. It is worth noting that the last interaction in the rankings obtained by signal-to-noise ratio analysis was not added to the input sources when the ANOVA was performed; for example, there was no interaction between C and D in the sources for the ANOVA on CT. As shown in Table 3, A, A × B, and B × C are highly significant (HS) factors with *p*-values less than 0.05.

**Figure 2:**
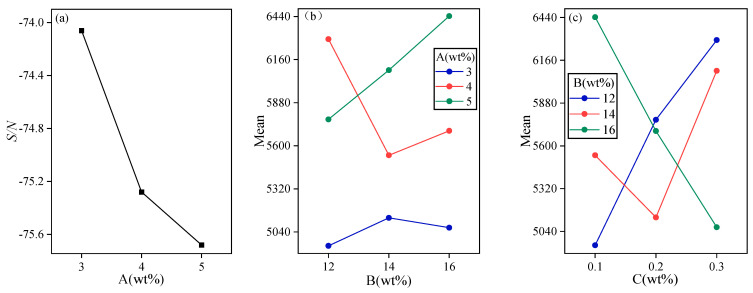
Important factors affecting the rate of cooling time (CT). (**a**) The signal-to-noise ratio curve graph of Na_2_SiO_3_·9H_2_O, (**b**) The interaction diagram of Na_2_SiO_3_·9H_2_O with KCl, (**c**) The interaction diagram of KCl with nano-α-Fe_2_O_3_.

Based on the values in Table 2 and combined with the S/N ratio and ANOVA, the effect of significant factors on the thermal conductivity is shown in Figure 2. As can be seen from the figure, the one-factor S/N plot represents the effect of each material content level; the interaction plot (also known as binary effect plot) is a plot of the true contribution of two factors to the characteristics when they act simultaneously, reflecting the effect of the interaction between the two factors. It should be noted that when there is a significant interaction between any two factors such as A and B, the optimal level of A and B should be chosen from the interaction of A and B, regardless of whether the effect of A or B itself is significant. From Figure 2a, it can be seen that the SMN content is the most critical factor affecting the cooling time (CT); SMN can determine the total mass of powder dissolved into the melt pool. Therefore, the cooling time (CT) decreases with the increase of SMN, which illustrates that the thermal conductive increases. The optimum level of SMN should be chosen from A1. By analyzing the interaction between A and B, and the interaction between B and C, based on Figure 2b,c, we consider that the interaction between A1 and B1, and B1 and C1, is the optimal parameter combinations. In conclusion, under the condition that the required time for heat conduction is relatively low, the optimal choices for the main factors are A1, B1, and C1.

#### 2.1.2. Supercooling Degree Analysis

The magnitude of the degree of supercooling (ΔT) is an important evaluation index of PCMs, which must be overcome in order for the material to effectively release heat energy [40]. The degree of supercooling as an important index for evaluating PCMs has been applied in a large number of literatures. The effect on the degree of supercooling (ΔT) at different modified material contents is shown in Table 4. The order of the effect of each factor on ΔT is A > D > B > A × D > A × B > C > A × C > B × C > B × D > C × D. From Table 5, it can be seen that A, B, D, and A × D were the highly significant (HS) factors, and A × B was the significant (S) factor.

**Figure 3:**
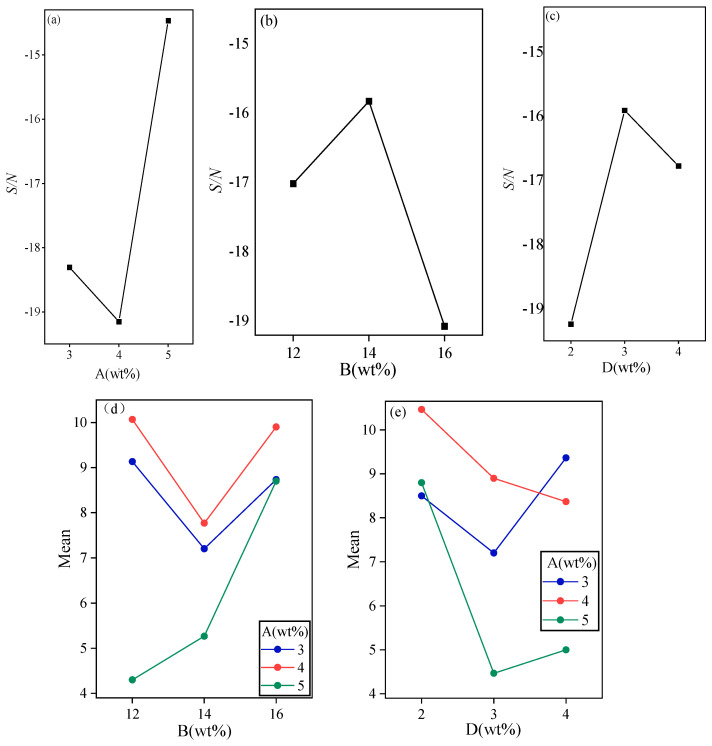
Important factors affecting the degree of supercooling. (**a**) The signal-to-noise ratio curve graph of Na_2_SiO_3_·9H_2_O, (**b**) The signal-to-noise ratio curve graph of KCl, (**c**) The signal-to-noise ratio curve graph of XG, (**d**) The interaction diagram of Na_2_SiO_3_·9H_2_O with KCl, (**e**) The interaction diagram of Na_2_SiO_3_·9H_2_O with XG.

The effects of A, B, and D on ΔT are very important, as shown in Figure 3. Among them, A is the most critical factor. It shows that it decreases and then increases, that is, the degree of supercooling decreases and then increases with the increase in content. This is due to the fact that the addition of SMN beyond a certain proportion not only fails to provide more nucleation sites, but also interferes with the crystallization process of DHPD, leading to an increase in ΔT [41]. From the effect of B, it can be found that the maximum supercooling of B can reach 14wt%, and higher or lower B is unfavorable to ΔT. Moreover, the effect of D on ΔT is also similar to the effect of B on ΔT, which may be due to the fact that DHPD loses its water of crystallization when it is heated to form anhydrous material or compounds with different degrees of hydration. The addition of KCl may affect this process, leading to a change in the temperature of release of the water of crystallization and thus affecting the degree of supercooling. Nano-α-Fe_2_O_3_ acts as a nucleating agent to reduce the degree of supercooling, but with the loss of water from the DHPD and the decrease in the concentration of the solution, ΔT increases again. From the interaction diagram, we know that the combination of A3 and B1, as well as A3 and D2, is the optimal parameters, and in summary, the optimal selection of the main factors is A3, B2, and D2 when the requirement for small ΔT is considered.

#### 2.1.3. Phase Transition Latent Heat Analysis

In fact, the latent heat of phase change (ΔH_m_) is the amount of heat absorbed or released per unit mass of material during the phase change process. The larger the latent heat value, the more heat the material can store or release during the phase change. The effects on the latent heat of phase transformation (ΔH_m_) at different modified material contents are shown in Table 6. The order of the effect of each factor on ΔH_m_ is A × B > C × D > D > B × C > A > A × D > B > A × C > C > B × D. As shown in Table 7, C × D is a significant (S) factor.

**Figure 4:**
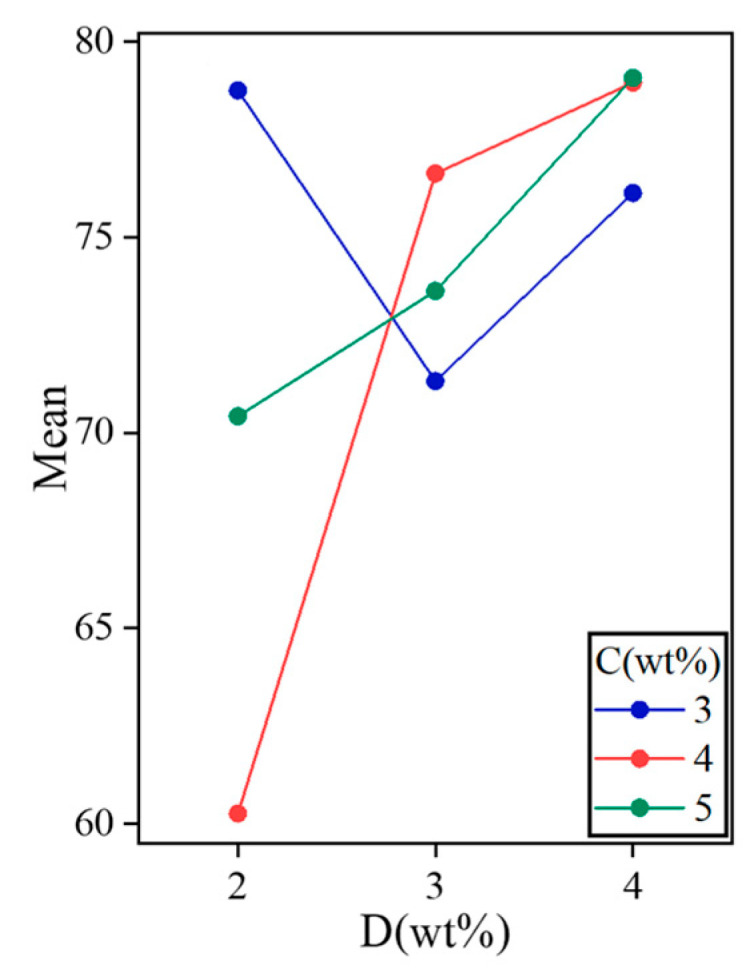
Important factors affecting the latent heat of phase transition.

According to the principle of higher latent heat of phase transition, as shown in Figure 4, when the content of C is 0.2 wt% and 0.3 wt%, the increase of B is favorable to the increase of ΔH_m_. XG, as a thickener, can effectively inhibit the phase separation phenomenon of DHPD. Phase separation leads to a decrease in the latent heat of phase transition of the material, and the addition of XG helps to maintain the homogeneity of the material, thus increasing the latent heat of phase transition. Nano-α-Fe_2_O_3_ addition also increases the thermal conductivity of the material. The increase in thermal conductivity helps the material to absorb and release heat faster during the phase transition, thus increasing the latent heat of phase transition. When both of them work together, a stable composite material may be formed, which makes the latent heat of phase transition higher. In summary, the optimal choices are D1C1, D3C2, and D3C3.

#### 2.1.4. Phase Transition Temperature Analysis

The phase transition temperature (T_m_) is a key parameter for the transition of substances between different phases, which has an important influence on the properties and applications of phase transition materials. The effects on phase transition temperature (T_m_) on different modified material contents are shown in Table 8. The order of the effects of each factor on T_m_ is B > B × C > D > C > A × B > B × D > A × C > C × D > A × D > A. As shown in Table 9, B is a highly significant (HS) factor and B × C is a significant (S) factor.

**Figure 5:**
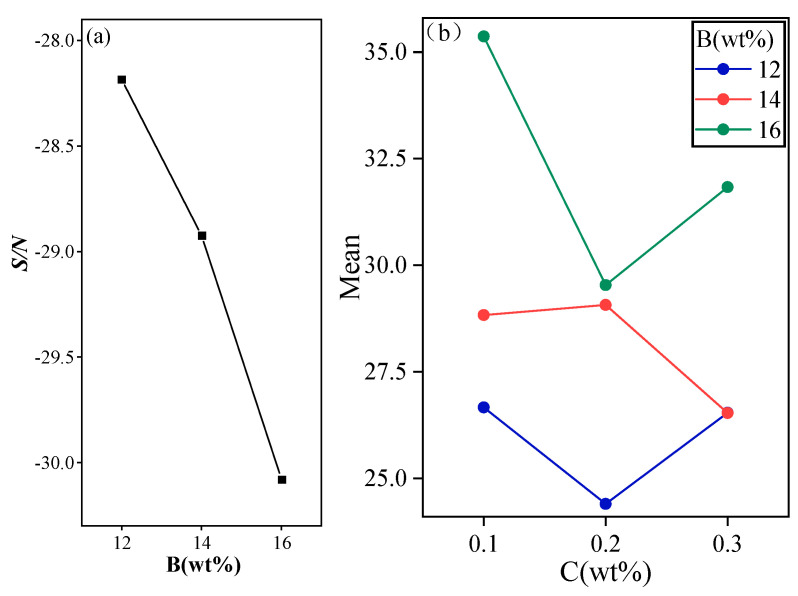
Important factors affecting the phase transition temperature. (**a**) The signal-to-noise ratio curve graph of KCl, (**b**) The interaction diagram of KCl with nano-α-Fe_2_O_3_.

B has the greatest effect on the characterization of the phase transition temperature (T_m_). As shown in Figure 5, the phase transition temperature (T_m_) decreases with the increase of KCl, which is due to the fact that the salt ions dissolved in water tend to weaken the attraction of the hydrogen bonds between water molecules, which decreases the freezing point of water and thus changes the phase transition temperature [40]. From the interaction of B and C, it can be seen that the phase transition temperatures of different contents of B are located in different stages, from which it can be further determined that B plays a decisive influence on the phase transition temperature. In summary, under the premise of low phase transition temperature requirement, the optimal selection of the main factors is B1 and C2.

Based on the above results and discussions, Table 10 summarizes the significant factors corresponding to each characteristic value and their trends. As can be seen from Table 10, the effects of different modified material contents on the performance characteristics of multivariate gel phase change materials are very complicated. Especially for the effect on ΔT, A and B are almost the most important parameters among all characteristics. In the selected range of levels, larger A has a negative effect on cooling time (CT) and larger B has a negative effect on Tm. Although B is not always important for each characteristic, the interaction effect with other factors plays an important role. In this paper, the effects of each feature are analyzed together. The C and D that can be selected from the results of each feature are C2 and D2, respectively. Based on the results of cooling time (CT), phase transition temperature and latent heat of phase transition measurements, the grade of B is determined to be B1. However, for the degree of supercooling, B2 should be selected. However, it is important to note that the effect of B on the phase transition temperature is very large. Therefore, the final grade of B was chosen as B1. The degree of supercooling had to be overcome in order for the material to efficiently release heat energy, so the value of A was A3. In summary, the optimal material content combinations were A3, B1, C1, and D2, that is, SMN 5 wt%, KCl 12 wt%, nano-α-Fe_2_O_3_ 0.2 wt%, and XG 3 wt%.

### 2.2. Physical and Chemical Structural Analysis

**Figure 6:**
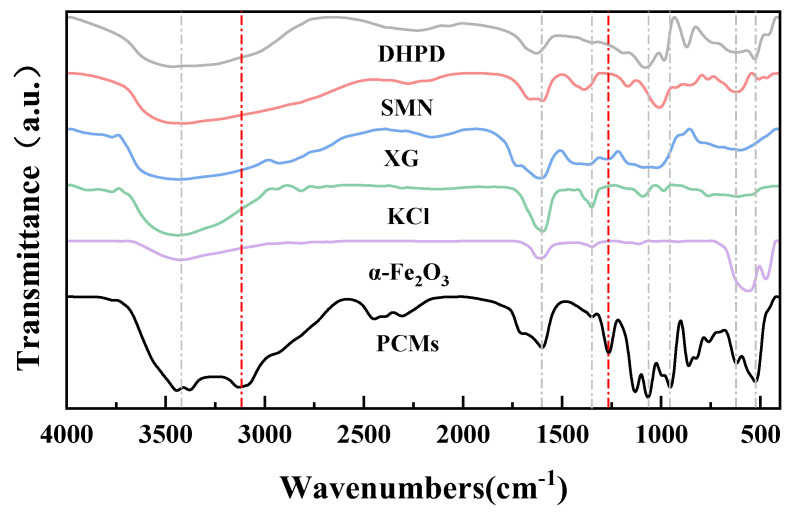
FTIR spectra of DHPD, SMN, XG, KCl, nano-α-Fe_2_O_3_, and PCMs.

The infrared absorption spectra of PCMs made of Nano-α-Fe_2_O_3_ with the four materials, DHPD, SMN, XG, and KCl, are shown in Figure 6. The detail distribution of typical bonds are listed in Table 11 [41,42,43,44] in which the peaks at 532 cm^−1^ and 630 cm^−1^ are attributed to the in-plane bending vibration of HPO_4_^2−^, the in-plane bending vibration of the C-H of the benzene ring in XG, and the reflection of infrared by the iron atoms. The absorption peaks at 960 cm^−1^ and 1060 cm^−1^ are asymmetric contraction vibrations of HPO_4_^2−^. The absorption peak at 1130 cm^−1^ is C-O in XG. The absorption peak at 1260 cm^−1^ is the in-plane bending vibration of O-H. The absorption peak at 1350 cm^−1^ is attributed to the bending vibration of -CH3 in XG. The absorption peak at 1600 cm^−1^ is attributed to the in-plane bending of SiO_3_^2−^ and the backbone vibration of the benzene ring in XG. The absorption peaks between 2300–2500 cm^−1^ are the sympathetic vibration of HPO_4_^2−^. A similar trend has been obvious in identifying the intensity of the characteristic bands with CPCMs [41]. The large broad peaks between 3000 and 3600 cm^−1^ are the stretching vibration of O-H. A peak at 1260 cm^−1^ in PCM sample is the anti-symmetric stretching vibration of HPO_4_^2−^ [42,43]. The 868.77 cm^−1^, 1071 cm^−1^, and 1137 cm^−1^ are the P-oh symmetric stretching vibration, P-O anti-symmetric stretching, and P=O symmetric stretching, respectively [44]. It is worth noting that KCl does not affect the infrared absorption peaks of the CPCM, and the absorption peaks of KCl at 1350 cm^−1^ and 1600 cm^−1^ are due to the lattice vibration, whereas KCl appears as an ionic form in CPCMs. The newly appeared absorption peak at 1260 cm^−1^ is attributed to the interaction of water molecules with the oxygen atoms on the surface of nano-α-Fe_2_O_3_ through hydrogen bonding; it also leads to the splitting of the large broad peaks between 3000–3600 cm^−1^. Other than that, the absorption peaks of PCMs are completely consistent with those of DHPD, SMN, XG, and nano-α-Fe_2_O_3_.

**Figure 7:**
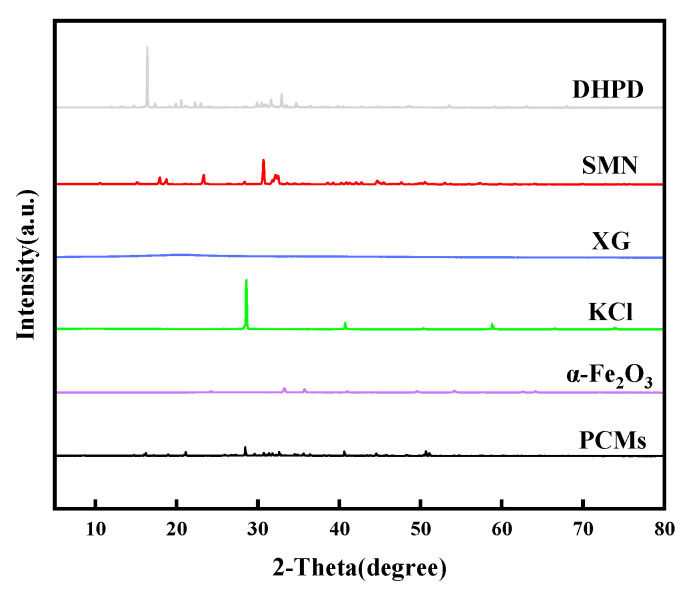
XRD patterns of DHPD, SMN, XG, KCl, nano-α-Fe_2_O_3_, and PCMs.

The XRD spectra of DHPD, SMN, XG, KCI, nano-α-Fe_2_O_3_, and PCMs are shown in Figure 7. The strong characteristic diffraction peaks of DHPD are located at 16.4°, 17.36°, 19.9°, 20.56°, 22.26°, 22.98°, 29.88°, 30.8°, 31.32°, 32.92°, 34.7°, and 53.2°. The diffraction peaks of SMN are at 15.14°, 17.94°, 18.74°, 23.34°, 30.66°, 32.14°, and 44.72°. The diffraction peaks of KCl are at 28.58°, 40.74°, and 58.8°; and the strong diffraction peaks of nano-α-Fe_2_O_3_ are located at 33.24°, and 35.76°. It can be seen that the XRD spectra of PCMs display the diffraction peaks of DHPD, SMN, KCl, and nano-α-Fe_2_O_3_, and there are no new obvious diffraction peaks, but the intensity of the peaks changes, which indicates that the PCMs have been successfully synthesized and no new substances are produced [45]. There is a broad diffraction peak at 20.68° with weak diffraction intensity, which indicates that XG has an amorphous structure. The PCM sample exhibited peaks in the 2-Theta range of 10–60°. The typical peak is located at 21.1°, 30.6°, 32°, 36.6°, 44.7°, and 50.9°, which is corresponding to (1,1,1), (−2,2,1), (2,2,1), (−2,3,1), (−1,5,1), and (1,6,0) plane, respectively [46]. 

**Figure 8:**
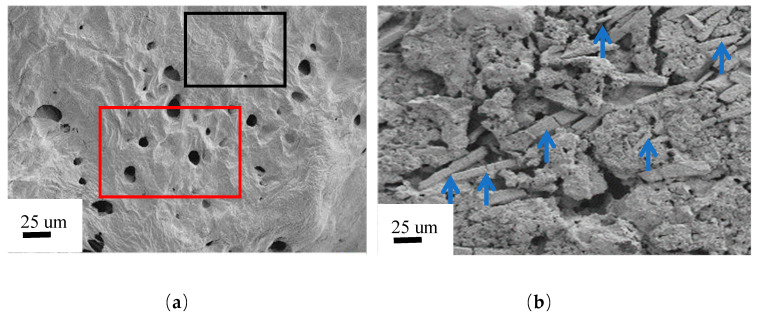
SEM images of: (**a**) DHPD and (**b**) PCMs.

Figure 8a is an SEM image of an unprocessed DHPD. A porous micro-structure is located in the red rectangle, and the regular structure is located in the black rectangle. Figure 8b is an SEM image of PCMs, which has become complex in structure with staggered distribution of particles of different shapes and sizes. As can be seen from Figure 8b, the various structures are relatively evenly distributed, and there are no obvious aggregation areas of components. The mixed materials form a more complex micro-structure through the interweaving of various components, which correspond to the Figure 6 and Figure 7. The respective characteristic typical bond of FTIR and diffraction peaks of XRD are confirming that the preparation of CPCM is simple physical overlay of four components. In addition, the individually crystallized Na_2_HPO_4_·12H_2_O is corrugated aggregation in somewhere. It may be caused by the surface energies on different planes of Na_2_HPO_4_·12H_2_O changing in the process of crystal growth, leading to the shape and size of crystals changing [47]. In conclusion, the phase change composite material developed in this paper can improve the performance of the original base phase change material without generating any new chemical components. Phase change composite materials have broad application prospects and value, especially in the energy storage of the walls of solar greenhouses.

## 3. Conclusions

Using the Taguchi design method, a multivariate phase change material modification experiment with Na_2_HPO_4_·12H_2_O as the starting material was designed. The experimental and analytical results show that the content of different modified materials plays a crucial role in the performance characteristics of multiple gel phase change materials. The contents of Na_2_SiO_3_·9H_2_O(A) and KCl(B) and the interaction B × C are important parameters influencing various characteristics. Within the selected range of levels, a larger A has a negative impact on cooling time (CT), and a larger B has a negative impact on T_m_. The optimal content of the modified materials of the multifunctional gel phase change materials are prepared by melt–blending method using Na_2_HPO_4_·12H_2_O as the starting material where 5 wt% of SMN, 12 wt% of KCl, 0.2 wt% of nano-α-Fe_2_O_3_, and 3 wt% of XG are used.

## 4. Materials and Methods

### 4.1. Materials

Na_2_HPO_4_·12H_2_O(DHPD, AR) was used as the PCM, Na_2_SiO_3_·9H_2_O(SMN, AR) was selected as the nucleating agent, and KCl(AR) was used as the phase transition temperature modifier, all of which were obtained from Sinopharm Chemical Reagent Co. (Shanghai, China). C_35_H_49_O_29_(XG, USP Grade) was chosen as the thickener and was supplied by Aladdin Chemical Co. Ltd. in Shanghai, China, and the thermal conductivity enhancer was α-Fe_2_O_3_, with an average particle size of 30 nm, from Shanghai Xinzhao Welding Materials Co. (Shanghai, China). All chemicals were used directly as received without further purification. The detailed amounts of different components were shown in Table 12 and Table 13. 

### 4.2. Composite Phase Change Material Preparation

**Figure 9:**
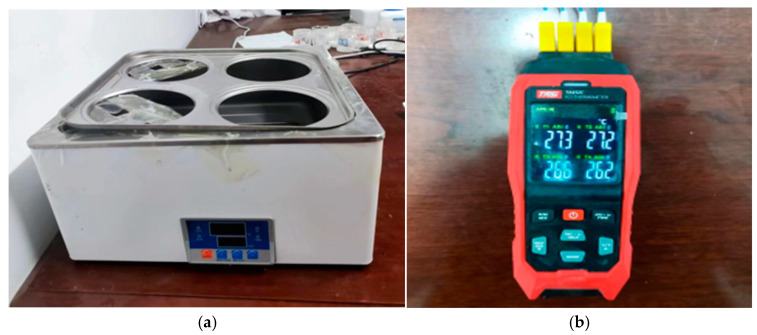
Experimental apparatus: (**a**) hermostat water bath; (**b**) real-time temperature recorder.

The preparation method of the DHPD multifunctional phase change material was the melt–blending method. First, the phase change material DHPD, nucleating agent SMN, phase change temperature regulator KCl, and thickening agent XG were weighed in different proportions in a beaker, stirred and mixed homogeneously with a glass rod at room temperature, and then sealed. After that, it is placed in a water bath at 60 °C for heating to melt completely. After complete melting, nano-α-Fe_2_O_3_ particles for thermal conductivity enhancement are added, and the mixture is thoroughly stirred with a glass rod. The heating is continued for 30 min and then taken out and left to cool naturally to obtain the gel phase change materials. The hermostat water bath (HH-4) and real-time temperature recorder (TA612C) are shown in Figure 9.

### 4.3. Experimental Method

In order to obtain gel phase change materials with low supercooling, suitable phase change temperature, high thermal conductivity and without phase separation, and to study the properties of multivariate gel phase change materials with different doping of materials, the experimental design was carried out by Taguchi design method, and four material parameters, SMN, XG, KCl, and nano-α-Fe_2_O_3_, were selected as the influencing factors, as shown in Table 12. In order to obtain the comprehensive effects of different proportions of modified material doping on the thermal properties of the multivariate gel phase change materials, and the four interactions of the four modified materials were added as influencing factors.

A L_27_(3^13^) Taguchi orthogonal design was performed using the statistical software MINITAB 21. The factors and their six interactions were arranged orthogonally according to the Taguchi design paradigm, and the detailed input doping parameters were listed in Table 13.

### 4.4. Methods of Analysis

The signal-to-noise ratio method was used to analyze the experimental data. The signal-to-noise ratio was a value used to measure the performance of a composite phase change material and evaluate the impact of each selected factor on it. The signal-to-noise ratio was analyzed based on three categories of quality characteristics: the higher the better (HB), the lower the better (LB), and the nominally best (NB), using Equations (1)–(3), respectively. Based on the ‘higher is better’ principle, the latent heat of phase transition was calculated. Based on the ‘lower is better’ principle, the supercooling degree, thermal conductivity, and phase transition temperature were calculated.

(1) $$\eta_{1}=-10\mathrm{lg}\left[\frac{1}{n}\sum_{k=1}^{n}\left(\frac{1}{y_{i}^{2}}\right)\right]$$

(2) $$\eta_{2}=-10\mathrm{lg}\left[\frac{1}{n}\sum_{k=1}^{n}\left(y_{i}^{2}\right)\right]$$

(3) $$\eta_{3}=-10\mathrm{lg}\left[\frac{1}{n}\sum_{k=1}^{n}\left(y_{i}^{2}-m\right)\right]$$

Here, η was the S/N ratio, n the number of repetitions of the experimental combination, $y_{i}$ was the experimental response, and m was the mean value of the specific response.

After calculating the S/N ratio, the experimental data obtained from Taguchi’s method were further processed by ANOVA, which allows for quantitative grading of the significance of the factors. The main purpose of ANOVA was to assess the bias in the experiment and to determine the relative bias and analyze the significance of the control factors using statistical techniques. The probability value (*p*-value) calculated by Minitab software was an important indicator of the degree of influence. When the *p*-value of a factor was less than 0.05, the effect of the factor was highly significant (HS); when the *p*-value of a factor was greater than 0.05 and less than 0.1, the effect of the factor was significant (S).

### 4.5. Test and Characterization Methods

**Figure 10:**
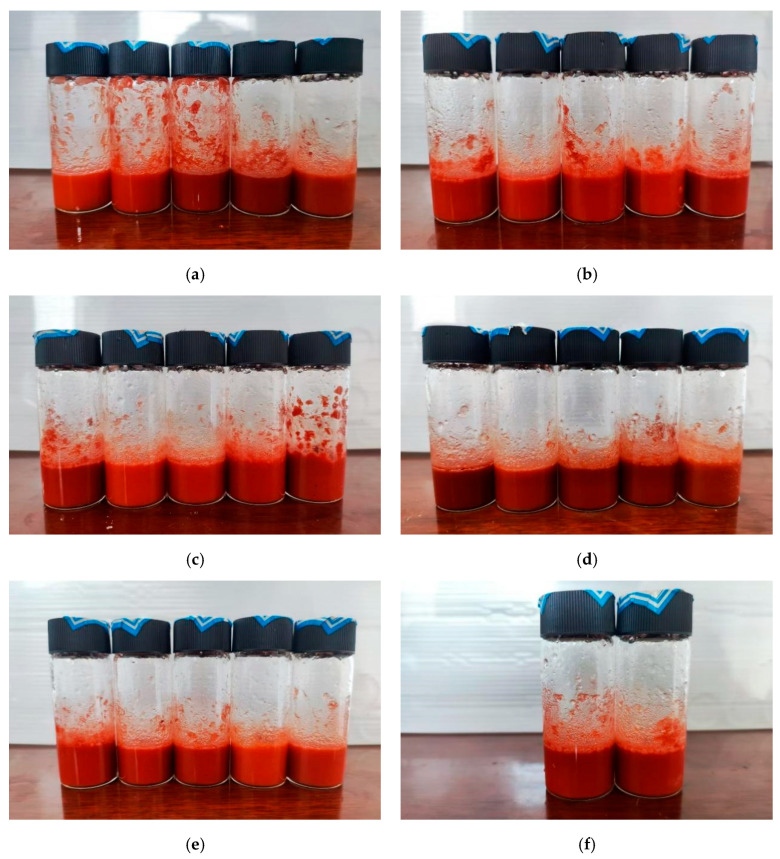
Front view of 27 test specimens: (**a**) samples of groups 1 to 5; (**b**) samples of groups 6 to 10; (**c**) samples of groups 11 to 15; (**d**) samples of groups 16 to 20; (**e**) samples of groups 21 to 25; (**f**) samples of groups 26 and 27.

Using a multiplexed data logger to draw the step–cooling curve, the sample was placed in the sample bottle and the temperature probe was inserted into it and the probe position did not touch the bottom and wall of the bottle, and after the sample was heated and melted in a constant temperature water bath at 60 °C, the sample bottle was put into a low-temperature thermostat pre-set at 10 °C; one temperature point was recorded every 10 s, and the degree of supercooling was calculated according to the graph of the change in temperature over time, and the time required for cooling down was selected from 50–15 °C. Then, the thermal conductivity was calculated. The experimental samples were shown in Figure 10. A differential scanning calorimeter (NETZSCH 214, DSC) was used to measure the thermal properties of PCMs. The scanning temperature range was 10–60 °C and the scanning rate was 5 °C/min. The microstructure of the gel phase change materials was investigated using a scanning electron microscope (ZEISS EVO 18, SEM). An infrared spectrometer (Bruker ALPHA II, FT-IR) was used to test the gel phase change materials, potassium bromide pressed tablets, with a wave number in the range of 4000–400 cm^−1^. An X-ray diffractometer (Rigaku Ultima IV, XRD) was used to characterize the crystalline properties of the gel phase change materials.

## Figures and Tables

**Table 1:** The experimental results.

No.	CT (s)	ΔH_m_ (J/g)	ΔT (°C)	T_m_ (°C)
1	5078	72.95	9.1	26.2
2	4940	60.51	8.7	27.2
3	4830	66.81	9.6	26.6
4	5550	80.88	6.8	34.2
5	4993	76.04	7.5	27.3
6	4852	64.35	7.3	25.7
7	5286	95.73	11	38.8
8	5083	72.13	9.1	31.7
9	4833	71.07	6.1	25.0
10	6608	62.68	10.8	20.9
11	5344	63.67	9.1	24.5
12	6927	78.73	10.3	34.3
13	5467	82.19	8.5	26.4
14	4955	87.42	5.3	34.6
15	6193	67.78	9.5	25.5
16	5724	65.29	9.5	25.9
17	6084	53.36	11.1	29.8
18	5284	70.53	9.1	32.9
19	5322	62.97	7.1	22.7
20	5933	78.46	2.6	25.5
21	6059	95.52	3.2	25.0
22	5654	86.14	2.7	24.1
23	5782	62.74	3.1	28.3
24	6838	76.46	10.0	27.2
25	6378	74.15	8.7	35.7
26	6290	95.52	9.3	38.2
27	6660	71.26	8.1	32.2

**Table 2:** Effect of different modified material contents on the rate of cooling time.

Level	A	B	A × B	C	A × C	B × C	D	A × D	B × D	C × D
1	−74.06	−75.01	−74.88	−74.97	−75.05	−75.25	−75.23	−74.98	−74.75	−75.25
2	−75.28	−74.89	−75.43	−74.83	−74.75	−75.23	−74.80	−75.26	−75.06	−74.83
3	−75.68	−75.12	−74.71	−75.23	−75.22	−74.54	−75.00	−74.79	−75.22	−74.94
Delta	1.63	0.23	0.72	0.39	0.47	0.71	0.43	0.48	0.48	0.42
Rank	1	10	2	9	6	3	7	5	4	8

**Table 3:** Thermal conductivity ANOVA.

Source	DoF	Adj SS	Adj MS	F-Value	*p*-Value	Significance
A	2	5,411,949	2,705,974	20.87	0.001	HS
B	2	100,032	50,016	0.39	0.692	
A × B	2	1,208,936	604,468	4.66	0.045	HS
C	2	368,486	184,243	4.12	0.296	
A × C	2	525,483	262,741	2.03	0.194	
B × C	2	1,242,587	621,293	4.79	0.043	HS
D	2	400,305	200,153	1.54	0.271	
A × D	2	429,452	214,726	1.66	0.250	
B × D	2	497,968	248,984	1.92	0.208	
Error	8	1,037,279	129,660			
Total	26	11,222,476				

**Table 4:** Effect of different modified material contents on the degree of supercooling.

Level	A	B	A × B	C	A × C	B × C	D	A × D	B × D	C × D
1	−18.31	−17.02	−17.30	−18.51	−17.77	−17.02	−19.24	−16.11	−16.81	−17.69
2	−19.15	−15.83	−18.65	−16.28	−16.39	−18.06	−15.91	−16.78	−17.98	−16.59
3	−14.47	−19.08	−15.97	−17.14	−17.76	−16.85	−16.78	−19.04	−17.13	−17.64
Delta	4.68	3.24	2.68	2.23	1.37	1.21	3.33	2.92	1.17	1.10
Rank	1	3	5	6	7	8	2	4	9	10

**Table 5:** ANOVA table of supercooling.

Source	DoF	Adj SS	Adj MS	F-Value	*p*-Value	Significance
A	2	47.656	23.828	11.76	0.004	HS
B	2	25.259	12.629	6.23	0.023	HS
A × B	2	13.907	6.954	3.43	0.084	S
C	2	9.034	4.517	2.23	0.170	
A × C	2	4.679	2.339	1.15	0.363	
B × C	2	3.932	1.966	0.97	0.420	
D	2	27.290	13.645	6.73	0.019	HS
A × D	2	18.003	9.001	4.44	0.050	HS
B × D	2	2.494	1.248	0.62	0.564	
Error	8	16.216	2.027			
Total	26	168.470				

**Table 6:** Effect of different modified material contents on the latent heat of phase transition.

Level	A	B	A × B	C	A × C	B × C	D	A × D	B × D	C × D
1	37.24	36.98	36.61	37.47	37.52	37.78	36.77	37.48	37.21	37.29
2	36.83	37.56	37.33	37.03	37.04	37.25	37.31	36.77	37.46	37.90
3	37.76	37.30	37.89	37.34	37.28	36.81	37.75	37.59	37.17	36.64
Delta	0.93	0.58	1.28	0.44	0.48	0.97	0.98	0.83	0.29	1.25
Rank	5	7	1	9	8	4	3	6	10	2

**Table 7:** Phase change latent heat ANOVA.

Source	DoF	Adj SS	Adj MS	F-Value	*p*-Value	Significance
A	2	288.16	144.08	1.48	0.285	
B	2	99.17	49.59	0.51	0.620	
A × B	2	539.58	269.79	2.76	0.122	
C	2	57.04	28.52	0.29	0.754	
A × C	2	62.11	31.05	0.32	0.736	
B × C	2	300.68	150.34	1.54	0.272	
D	2	306.14	153.07	1.57	0.266	
A × D	2	230.78	115.39	1.18	0.355	
C × D	2	611.47	305.73	3.13	0.099	S
Error	8	780.81	97.60			
Total	26	3275.93				

**Table 8:** Effect of different modified material contents on phase transition temperature.

Level	A	B	A × B	C	A × C	B × C	D	A × D	B × D	C × D
1	−29.21	−28.18	−28.78	−29.53	−28.86	−29.87	−28.67	−29.14	−29.02	−29.06
2	−28.93	−28.92	−29.48	−28.77	−29.37	−28.59	−28.87	−28.82	−29.39	−29.27
3	−29.05	−30.08	−28.92	−28.89	−28.96	−28.72	−29.64	−29.22	−28.77	−28.85
Delta	0.29	1.89	0.70	0.76	0.51	1.27	0.97	0.40	0.62	0.42
Rank	10	1	5	4	7	2	3	9	6	8

**Table 9:** Phase change temperature ANOVA.

Source	DoF	Adj SS	Adj MS	F-Value	*p*-Value	Significance
A	2	3.469	1.734	0.10	0.903	
B	2	187.447	93.723	5.58	0.030	HS
A × B	2	36.329	18.164	1.08	0.384	
C	2	33.609	16.804	1.00	0.410	
A × C	2	11.496	5.748	0.34	0.720	
B × C	2	106.336	53.168	3.16	0.097	S
D	2	53.149	26.574	1.58	0.264	
B × D	2	20.180	10.090	0.60	0.571	
C × D	2	14.196	7.098	0.42	0.669	
Error	8	134.398	16.800			
Total	26	600.607				

**Table 10:** The main factors affecting the performance characteristics of multivariate gel phase change materials and their changing trends.

Ranking#Parameters	CT	ΔT	ΔH_m_	T_m_
1	A, −ve	A, ±ve	C × D	B, −ve
2	A × B	B, ±ve	-	B × C
3	B × C	D, ±ve	-	-
4	-	A × B	-	-
5	-	A × D	-	-

+ve positive, −ve negative, ±ve positive/negative.

**Table 11:** Typical bands of different components [41,42,43,44].

No.	Peak Location	Component
1	532 cm^−1^	HPO_4_^2−^, C-H, XG
2	630 cm^−1^	HPO_4_^2−^, C-H, XG
3	960 cm^−1^	HPO_4_^2−^
4	1060 cm^−1^	HPO_4_^2−^
5	1130 cm^−1^	C-O
6	1260 cm^−1^	O-H
7	1350 cm^−1^	-CH_3_
8	1600 cm^−1^	SiO_3_^2−^

**Table 12:** Factors and amounts of orthogonal tests.

Level	Element			
	A Na_2_SiO_3_·9H_2_O	B KCI	C Nano-α-Fe_2_O_3_	D XG
	(wt%)	(wt%)	(wt%)	(wt%)
1	3	12	0.1	2
2	4	14	0.2	3
3	5	16	0.3	4

**Table 13:** Experiment L_27_(3^13^) Taguchi hybrid array design.

No.	A	B	A × B	C	A × C	B × C	Blank Column	D	A × D	B × D	Blank Column	C × D	Blank Column
1	3	12	1	0.1	1	1	1	2	1	1	1	1	1
2	3	12	1	0.1	2	2	2	3	2	2	2	2	2
3	3	12	1	0.1	3	3	3	4	3	3	3	3	3
4	3	14	2	0.2	1	1	1	3	2	2	3	3	3
5	3	14	2	0.2	2	2	2	4	3	3	1	1	1
6	3	14	2	0.2	3	3	3	2	1	1	2	2	2
7	3	16	3	0.3	1	1	1	4	3	3	2	2	2
8	3	16	3	0.3	2	2	2	2	1	1	3	3	3
9	3	16	3	0.3	3	3	3	3	2	2	1	1	1
10	4	12	2	0.3	1	2	3	2	2	3	1	2	3
11	4	12	2	0.3	2	3	1	3	3	1	2	3	1
12	4	12	2	0.3	3	1	2	4	1	2	3	1	2
13	4	14	3	0.1	1	2	3	3	3	1	3	1	2
14	4	14	3	0.1	2	3	1	4	1	2	1	2	3
15	4	14	3	0.1	3	1	2	2	2	3	2	3	1
16	4	16	1	0.2	1	2	3	4	1	2	2	3	1
17	4	16	1	0.2	2	3	1	2	2	3	3	1	2
18	4	16	1	0.2	3	1	2	3	3	1	1	2	3
19	5	12	3	0.2	1	3	2	2	3	2	1	3	2
20	5	12	3	0.2	2	1	3	3	1	3	2	1	3
21	5	12	3	0.2	3	2	1	4	2	1	3	2	1
22	5	14	1	0.3	1	3	2	3	1	3	3	2	1
23	5	14	1	0.3	2	1	3	4	2	1	1	3	2
24	5	14	1	0.3	3	2	1	2	3	2	2	1	3
25	5	16	2	0.1	1	3	2	4	2	1	2	1	3
26	5	16	2	0.1	2	1	3	2	3	2	3	2	1
27	5	16	2	0.1	3	2	1	3	1	3	1	3	2

## Data Availability

The data sets presented in this study are available within the article.
